# Clinical Remarks on Sciatic Neuralgia, with a Case

**Published:** 1861-03

**Authors:** Joseph Pancoast

**Affiliations:** Attending Surgeon; Professor of Anatomy in Jefferson Medical College


### SELECTIONS.

*Clinical Remarks on Sciatic Neuralgia, with a Case. Delivered at the Pennsylvunia Hospital, Jan. 16th,* 1861. By JOSEPH PANCOAST, M D., *attending Surgeon; Professor of Anatomy in Jefferson Medical College.*

The case before us is one of sciatic neuralgia in an adult male, which the patient attributes to his lifting a heavy stove. If this be the case, it must have been from the immense pressure of the abdominal muscles and the diaphragm, acting together, in the violent effort of lifting, and squeezing all the viscera between them, and thus pressing upon the sciatic plexus of nerves where it passes out at the upper sciatic notch.

Cases of sciatic neuralgia are always difficult to cure. Their causes are various; sometimes it depends upon a growth of bone, a little exostosis pressing upon some one of the branches of the great sciatic plexus of nerves, while at other times, as in neuralgia of other parts, it depends upon an inflammation of the neurilemma of the nerve itself.

Dr. Physic believed that, in these cases, an effusion of serum occurred in the sheath; but this has not yet been revealed by anatomical observation.

Very many cases of sciatica may be traced to mechanical causes of pressure. Whenever patients complain of excruciating pains down the leg, and at the same time in the back, so that the course of the sciatic nerve can be traced out, the difficulty will very frequently be found to depend upon the accumulation of fæces in the colon or in the rectum, pressing upod the branches of the sciatic nerve, and of course, in these cases, a cure may often be afiected in twenty-four hours by a thorough purge of one or two drops of Croton oil, with four grains of rhubarb.

Sometimes, however, in obstinate cases of sciatica, even after death, it is impossible to find any real physical cause for the suffering. It seems, then, to be often owing to a derangement of the whole nervous system.

As the pathology is obscure in these cases, the treatment is more or less empirical. The patient before us has been suffering for thirteen months, six of which have been passed in the hospital; various plans of relief have been instituted without the slightest advantage. He walks with pain and difficulty, using a cane and limping along. He has taken a dose of Croton oil; but, though causing free purgation, it has not benefited him. He complains of great pain along the peroneal nerve.

Where there is pain along a particular nerve, and limited to one spot, it is generally gouty, rheumatic, or syphilitic, as these diseases affect the fibrous tissues, which swell and compress the nerve.

If there be reason to suspect a syphilitic origin to the neuralgia, then the treatment would consist in giving mercury in the form of calomel, with a little James's powder and some morphia, until salivation is produced.

Neuralgia about the face is thus frequently relieved when dependent on syphilis, which is often the case, as the nervous branches are numerous, and pass through different foramina lined with periosteum, the slightest affection of which causes it to press upon the nerves in contact with it. In other cases, the neuralgia, especially that form known as *tic douleureux* arises from malaria. When it occurs from miasmata, it is cured by quinine, combined with morphia.

So little evidence of disease does this patient before us present, that there has been some suspicion that he feigned to be worse than was really the case, in order to retain his comfortable quarters for the winter; but this suspicion is probably groundless.

Various methods have been employed to procure relief, but without avail. Counter-irritation has been made in various ways; small blisters have been placed up and down the course of the leg, which seem to have done more good than anything else. Sometimes large doses of the spirits of turpentine are given with very great advantage.

Many cases have been cured by touching a hot iron between the little toe and the toe next to it, until the part is sore. Malgaigne recommends touching the helix of the ear in the same manner. This, he says, will often produce an instantaneous cure from the general shock to the nervous system. So with other remedies.

In these cases it is necessory to examine the hip joint, to see whether there exists any sign of disease.

In some of these cases, the greatest benefit has been derived from acupuncturation, a practice which originated with the Chinese and Japanese.

Formerly, Dr. Pancoast was in the habit of passing needles through with great care, so as not to strike the nerve; now he prefers to run the needles directly through the nerve and pin it down upon the ischium.

The needles, should be inserted by rotation, separating the tissues, for they will not pass through the fibres, but separate them and pass between. A needle might be passed through a great vessel without doing damage, and through the heart without serious consequences. When an acupuncture needle is pasted through a nerve, it often gives rise to a feeling of heat or a glow of warmth through the part to which the nerve is distributed.

Dr. Pancoast has seen cases of neuralgia of the scalp in which death would have ensued, but from the adoption of this process, and he has passed as many as eighteen or twenty at one operation, driving the pins as far as possible into the bones of the scull, producing perfect relief in the course of a few minutes.

In the present case, the acupuncture needles were passed at small distances through the nerve into the periosteum covering the ischium, pinning the sciatic nerve to the tuberosity of the ischium. The needles are to be left in for some time.

January 23d. It is now certain that injustice was done the patient by the suspicion of feigning the disease. As he walks he presses the outer border of the foot upon the ground, raising the inner border, though, by a straining effort, he can succeed in bringing the inner border to the ground. This is owing to a neuralgic condition of the cutaneous nerves. The metatarsal bone of the little toe is pressed down upon the floor as he walks.

Since the acupuncturation he has had no pain about the sciatic notch ; but otherwise he is not improved, and he walks no better than before.

When asked to point out the seat of pain, he traces up distinctly the cutaneous branch of the musculo-cutaneous nerve, a division of the peroneal in the ham going up to the head of the fibula.

There is no locality in the body where the neuropathic condition is more difficult to remove than in this branch, which winds around the head of the bone, and, passing sometimes underneath one of the peronei muscles, winding around the posterior portion of the exterior ankle bone, while another branch winds around the foot.

Perhaps it is to avoid the motion of these muscles that he walks with his foot on its outer border.

Dr. Pancoast has sometimes found neuralgia, involving this nerve, utterly beyond control. He has tried the hot iron, blisters, the endermic use of morphia and of strychnia, without much benefit, and it is not a nerve which we dare to divide, because such proceedure would be followed by paralysis of the peronei muscles, as well as of sensation of the outer margin of the foot ; it would produce dropping of the toe, in this effect analagous to the musculo-spiral nerve of the arm.

It is proposed to try the effect of a number of moxas along the course of the sciatic nerve, and if benefit should result from this treatment, to keep up a succession of blisters.

The hot iron of Carrigan has already been applied, but without benefit.

Pressure over the sciatic notch produces no longer pain, though it made him wince, before he was subjected to acupuncturation.

The moax is preferable in this case to the hot iron, because it burns more slowly and thereby makes a longer and deeper impression.

A quantity of lint, wet with warm water, was laid about the part selected for the application of the moxas, in order to protect the surrounding skin from the scintillation.

The moxas employed were made of gun cotton which had been soaked in a solution of the chlorate of potash, and allowed to dry completely ; the cotton being then rolled up into rounded masses and fastened in a cylindrical form by a piece of linen wrapt round them.

Moxas may be made of other material ; they are made from the pith of the sun-flower plant ; of pledgets of old linen ; of rotten wood completely dry and powdered and mixed with alcohol, rolling the mass into a cylindrical form, which was the favorite moxa of Baron Larry.

It was not desirable, in the present instance, to administer an anæsthetic, because, in order to judge of the impression made upon the nervous system by the application, it is necessary to retain the sensation of the patient.

Three moxas were applied along the course of the nerve; one where it winds around the ankle and the others higher on the limb. Immediately after the moxas had ceased burning, the parts around were washed with strong aqua ammonia as a contra-stimulus.

The day following the application of the moxas, the man was able to walk about the ward with very little difficulty and considered himself almost well. The day after, however, he began to relapse a little, and the next day he did not appear to walk much better than he did before the moxas were applied, though he places the foot more firmly on the ground.

His condition is much better in the morning than later in the day.

There is no local specific disease in these cases. It is a general affection of the nervous system, which manifests itself in some particular nerve. It is quite different from the sciatica, which sometimes comes from some affection of the neurilemma of the nerve, or from syphilitic or other disease of the periosteum, which causes pressure upon the nerve.

When the hot iron is applied in these cases, as recommended by Malgaigne, it is not with the hope of benefit from the local effects of the remedy, but it is with the hope that the shock produced upon the whole mass of the nervous system will produce relief.

The patient has had no return of the pain about the sciatic notch, or at the lower part of the limb; but it still remains about the femoral portion of the sciatic nerve, to which some more moxas will be applied.—*Med. and Surg. Reporter.*

